# Use of insecticide-treated nets (ITNs) following a malaria education intervention in Piron, Mali: a control trial with systematic allocation of households

**DOI:** 10.1186/1475-2875-4-35

**Published:** 2005-07-25

**Authors:** Michelle Rhee, Mahamadou Sissoko, Sharon Perry, Willi McFarland, Julie Parsonnet, Ogobara Doumbo

**Affiliations:** 1 Health Research and Policy Department, Stanford Medical School, Stanford University, Stanford, CA 94305, USA; 2 Malaria Research and Training Centre, Department of Epidemiology of Parasitic Diseases, Faculty of Medicine, Pharmacy and Odonto-Stomatology, University of Bamako Mali, P.O. Box 1805, Bamako, Mali; 3 San Francisco Department of Public Health, Suite 500, 25 Van Ness Avenue, San Francisco, CA 94102, USA

## Abstract

**Background:**

Insecticide-treated nets (ITNs) reduce malaria morbidity and mortality, but use is limited. A barrier to ITN use may be lack of knowledge regarding malaria transmission and prevention. This study is a controlled trial comparing ITN use and malaria knowledge levels between households in Piron, Mali, undertaken in 2003.

**Methods:**

Households received net impregnation services either with or without antecedent education. The main outcome measure was ITN use, defined as impregnation of at least one of the household's existing bednets with insecticide during the study. Knowledge about malaria and prevention practices was assessed pre- and post- educational intervention. Results were analysed by household and by individual.

**Results:**

Forty-nine percent (34/70) of households who received the educational component impregnated their nets in comparison to 35% (22/62) of households who did not (OR = 1.6 CI = 0.8–3.3, P = 0.19). In individual analysis, ITN use was significantly greater in participants who had received the educational intervention (48%) vs. individuals who did not (33%, OR = 1.9, P = 0.012). Knowledge levels about malaria significantly increased for each individual pre- versus post- educational intervention (average change score = 2.13, standard deviation = 1.97, t = -17.78, P < 0.001), although there was no difference found between educational (change score = 2.14) and control groups (change score = 2.12).

**Conclusion:**

It is possible to educate individuals about malaria and to implement net impregnation services with limited resources. Greater accessibility to net-impregnation services is necessary but not sufficient to increase ITN use.

## Introduction

A number of studies have demonstrated that the use of insecticide-treated nets (ITNs) is effective in reducing malaria-related morbidity and mortality [1-6]. A 25% reduction in all-cause mortality for children one to nine years of age was detected during the first year of the Gambian National Bednet Program [2]. In Kilifi District, Kenya, a 33% reduction in mortality and a 44% reduction in hospital admissions for severe malaria were also found [5].

Although these trials have demonstrated that ITNs are an effective malaria control strategy, there have been many challenges to ITN distribution, acceptance and utilization when trying to implement large-scale ITN programs [2]. Knowledge about the cause of malaria and about the existence of ITNs was low in many malaria-endemic communities [7-9]. For those areas which have been reached by publicity campaigns, high cost and lack of access were some of the reasons stated as to why ITNs were not used [10-12].

Since 1993, one of the Malian National Malaria Control Program's (NMCP) main objectives was to have 90% of net users in Mopti region treating their bednets with insecticide, but it has only achieved 10–30% usage rates to date (NMCP, unpublished data). In 2000, a household survey was conducted in four villages of Mopti region in order to identify the barriers to ITN use. Although a government media campaign about ITNs reached all villages, knowledge about malaria and about the benefits of ITN use was highly variable among the four villages. Households treating their nets with insecticide had significantly higher levels of knowledge about malaria and its prevention [13]. Reasons why people did not impregnate their bednets included: not knowing anything about ITNs, cost, and not having net impregnation services readily available in the village. In the village of Piron, ten of 73 households stated that they had previously treated their bednets and had seen the benefits of ITNs but were not retreating their nets, because there were no net treatment services available in close proximity to their households.

Based on these findings, a net-impregnation service was installed within Piron run by the community itself. An antecedent household-level educational program that promoted ITNs by relating use with malaria prevention was also implemented for half the village households during the study period. The objectives of this study were to measure the impact of education plus service availability on the level of knowledge about malaria and on ITN use.

## Materials and methods

### Study Design

**Figure 1:**
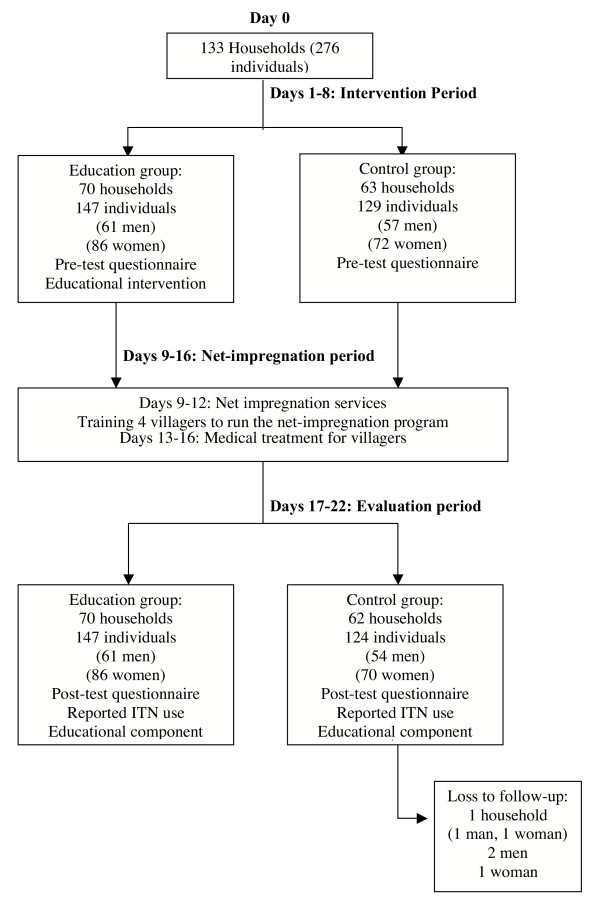
Study design and timeline.

This was a controlled intervention study performed in Piron, Mali of the effects of education on ITN use, as defined by impregnation of at least one of the households' existing bednets with insecticide, and malaria knowledge. Briefly, pre-test questionnaires were administered to 133 households during the intervention period. Households were then systematically assigned to receive either an educational component or not. All households were then offered the chance to have their nets impregnated by village agent trainees during the net-treatment campaign period. Lastly, individuals were given a post-test questionnaire during the evaluation period to measure ITN use and change in knowledge about malaria. The study design is shown Figure 1.

### Setting

This study was conducted during July-August 2003 in Piron village located in Bandiagara District, Mopti region, a sahelian area where malaria is endemic. Malaria prevalence rates for Piron, measured during the dry season for children ages one to nine years, was 61% (Keita *et al*., personal communication), but transmission rates are much higher at the end of the rainy season in September-October. Piron has no electricity or running water and no health facilities are available within the village. Although Piron is only 17 kilometres away from Goundaka – the closest village with health and net impregnation facilities – the roads are impassable during the rainy season and very few individuals have a method of transportation other than walking.

### Study design

#### Sampling design

A complete village sample of adults (estimated total population 1000, with 300 adults) was carried out with the household as the unit of allocation. To be eligible for the household survey, subjects had to have children. The Mopti Region Health Information System estimated 150 households in Piron, although only 133 were present at the time of study. Every second household was systematically assigned in equal proportions to receive the educational intervention with net-treatment services (study group) or net-treatment services alone (control group).

The protocol was reviewed and approved by the Administrative Panel for Human Subjects in Medical Research at Stanford University in the United States and the Institutional Ethical Committee at the Faculty of Medicine, Pharmacy and Odonto-stomatology, University of Bamako, in Mali. Informed consent was obtained from the village leaders and all individuals participating in the study. All individuals were given standard treatment for simple illnesses regardless of their participation in the study.

#### Intervention period (Days 1–8)

Two trained field guides, along with two local village guides who introduced them to households as is customary, went door-to-door to orally administer the pre-intervention questionnaires in the local languages to consenting individuals. Questionnaires were first developed in English, translated into French and then into Peul or Dogon, and back-translated to French and then English for verification.

Ten pre-test questions assessed individuals' knowledge of malaria, including ability to define malaria, to recognise signs and symptoms of the disease and knowledge of transmission, susceptibility, prevention and treatment. Additional information solicited in the one-on-one, in-depth interviews included individuals' demographic information, including socio-economic status (SES), and specific malaria prevention practices used such as ITNs, bednets, sprays and mosquito coils.

The intervention consisted of education about signs and symptoms of malaria, susceptibility, transmission and prevention of malaria and information about the benefits of ITN use, including, how, when and where to impregnate nets. Husband and wife/wives from each household assigned to the education group were educated together as a unit immediately after pre-test questionnaires were administered. Households in the control group received no antecedent education. All households were given the opportunity to have their nets treated at the end of the intervention study period, during the establishment of permanent net-impregnation services within the village. To the extent possible, interviewers were blinded to group assignment by reassigning interviewers during the post-education evaluation period.

#### Net-impregnation period (Days 9–16)

A community-based skills training format [14] was used to train four members of the community to run the net-impregnation program within their village. They were educated on the following parameters: malaria transmission and prevention, how to treat bednets with insecticide and how to use a self-monitoring system to collect data on how many nets are treated, how often and who uses them. They were also trained in recognising signs and symptoms of simple malaria and correct treatment dosages for different age groups.

During the training period, nets were impregnated by the village agent trainees for an at-cost price of 200 CFAF (1 US$ = 560 francs de la Communauté Financière d'Afrique [CFAF] at the time of study) per net or curtain regardless of size. Because cost was previously identified as a major inhibiting factor to using ITNs, the minimal price was charged that would permit purchase of more insecticide from the Mopti Regional Health Centre, thus enhancing long-term sustainability. At the end of the study, a village meeting was held to discuss the parameters of the program, namely the toxicity of the permethrin and the price of net impregnation they must charge in order to keep the program operating.

#### Evaluation period (Days 17–22)

The primary outcome measure was ITN use, as defined by impregnation of at least one of the households' existing bednets with insecticide, at the end of the study period. Change in knowledge about malaria transmission and prevention was assessed by comparing responses to questions pre- and post-intervention. After the post-test evaluation survey, control households received the educational component.

### Statistical analysis

Data were analysed with SPSS (Version 11.5). 'Intent-to-treat' analysis assessed the success of the educational intervention by measuring the ITN use for households in the intervention versus control arms using logistic regression analysis with household as the unit of allocation, weighting responses according to the number of individuals within each household who participated in the study. The weighting scheme took into account that there can be one or more household decision-makers, that everyone in the household had equal access to ITNs and that some household members may sleep under a single bednet. In a separate analysis, ITN use was analysed by individual, that is, by 'treatment-per-protocol' logistic regression analysis, under the rationale that households typically had more than one bednet and because education uptake was highly individualised. Nonetheless, men and women were educated as a household unit.

In determining change in knowledge, each of ten interview questions pertaining to malaria disease, symptoms, transmission, and prevention were assigned a point value of "1" for each correct answer and "0" for an incorrect answer to questions with only one response or "-1" for an incorrect answer to questions with greater than one response and summed for each respondent. This method of scoring responses produced a normal distribution when assessing the change in knowledge for individuals from baseline to post-intervention surveys. The paired t-test was used to measure change in pre- and post-test scores within subject. Mean change in household knowledge levels between control subjects and subjects receiving the educational component were also weighted according to household numbers in linear regression analysis.

All regression analyses were adjusted for non-randomized characteristics; that is, the date when individuals received the educational component, which field worker conducted the education and baseline characteristics which were significantly different between intervention and control groups. Beta values (B), odds ratios (OR), adjusted odds ratios (AOR) and 95% confidence intervals (CI) are presented.

## Results

### Demographic characteristics

In July 2003, 133 households (276 individuals) underwent systematic allocation: 70 households (147 individuals, 61 men and 86 women) to receive an educational component with the offer to treat their bednets and 63 households (129 individuals, 57 men and 72 women) to receive the offer of net-impregnation services alone (Figure 1). Of those approached, there was a 100% participation rate. No one crossed over between groups. Five individuals from the control group were lost to follow-up: a husband and wife from one household, and two men and one woman from separate households (62 households = 124 individuals, 54 men and 70 women). The 'intention-to-treat' and 'treatment-per-protocol' analysis was based on the remaining 132 households with one to four responders per household and 271 individuals, respectively.

Most baseline characteristics were similar in the two groups (Table 1). The mean age was 40 years and ages ranged from 14–80 in both groups. The individuals were Muslim (100%) and were primarily Dogon ethnicity (82–84%) with the other 15–18% being a mixture of Peul, Sonnike, Bambara and Maraka (primarily Peul). All residents were subsistence-level agriculturists, with less than 40% of both groups having another form of work for monetary income. Less than 15% of the population was literate, with slightly more than half of that percentage being literate in French. There was a significant difference in previous ITN use (education = 13% vs. control = 2%, P = 0.02) and median number of deceased children (education = 1 vs. control = 2, P = 0.014) between the two groups. SES, whether measured as earning income or possessions, was balanced across education and control groups.

**Table 1:** Baseline demographic characteristics of individuals.*

**Household characteristics**	**Education group ** **(N = 70)**	**Control group ** **(N = 62)**	**P Value**
Total individuals- no.	147	124	
Women- no. (%)	86 (58.5)	70 (56.5)	0.73
Men- no. (%)	61 (41.5)	54 (43.5)	
Age-years	40.4 ± 13.5	40.3 ± 13.7	0.99
Ethnicity- no. (%)			
Dogon	124 (84)	102 (82)	0.22
Peul	18 (12)	12 (10)	
Other	5 (3)	10 (8)	

Median number of responders per household	2	2	

Household size- median (25 and 75 quartiles)	5 (4, 8)	5 (4, 8)	0.84
Number of children total- median (25 and 75 quartiles)	5 (2, 7)	5 (3, 8)	0.16
Living- median (25 and 75 quartiles)	3 (2, 5)	3 (2, 5)	0.78
**Deceased- median (25 and 75 quartiles)**	**1 (0, 3)**	**2 (1, 4)**	**0.014**
At least one responder is literate- no. (%)†	15 (21)	11 (18)	0.60
At least one responder is a trader- no. (%)‡	13 (19)	17 (27)	0.23
At least one responder earns an income- no. (%)	24 (34)	24 (39)	0.60
Socio-economic status (SES) §	0.9 ± 1.6	0.8 ± 1.1	0.53

Malaria prevention methods used currently- no. (%)¶			
**ITNs used previously**	**9 (13)**	**1 (2)**	**0.02**
Untreated nets	69 (99)	57 (92)	0.10
Insecticide sprays	6 (9)	5 (8)	0.92
Mosquito coils	12 (17)	9 (15)	0.68

*Plus-minus values are means ± standard deviations (SD).

† Literate in any language, having studied for at least one year.

‡ All individuals were subsistence-level farmers.

§SES was calculated based on the number of material possessions. Each item was weighted according to their market value. SES value of 1 is approximately equal to 135,000 CFA ($241).

¶Percentages represent households who had at least one individual using stated method. Most stated that they used these methods against the nuisance of mosquitoes, not for malaria. None of the households were using ITNs at the time of study.

### ITN use

At baseline interview, none of the 132 households were using ITNs. The most common reasons for not treating their nets were cost (59%), availability (23%) and lack of knowledge regarding the effectiveness of ITNs in preventing malaria (11%). However, 93% of those who did not treat their nets during the study stated that cost was the main reason. Other malaria prevention methods are summarised in Table 1, with untreated bednets being the most common malaria prevention method (96%).

Forty-two percent (56/132) of households impregnated at least one of their nets with insecticide during the study. Forty-nine percent (34/70) of households who received the educational component impregnated their nets in comparison to 35% (22/62) of households who did not, although this difference was not statistically significant (OR = 1.6 CI = 0.8–3.3, P = 0.19). When stratified by day of pre-test interview, however, households in the intervention group who were interviewed during the first two days were significantly more likely to impregnate their nets than control group households (OR = 12.67, CI = 1.18–135.96, P = 0.04). When the household analysis was adjusted for significantly different baseline variables, none of them influenced the effect of the education intervention on ITN use.

Households in the education group tended to impregnate more nets per household (N = 1.01) than those in the control group (N = 0.66, t = 1.90, P = 0.059). Thus when analysed by individual, 48% (71/147) versus 33% (41/124) of individuals in education and control groups, respectively, impregnated their nets (unadjusted OR = 1.9, CI = 1.15–3.10, P = 0.012). Individuals assigned to the education group were still significantly more likely to impregnate their bednets than control group individuals when analysis was adjusted for day of baseline interview and pre-test interviewer. Interview day was significantly associated with ITN use independently of the educational intervention (AOR = 0.52, CI = 0.40–0.67, P < 0.001) as was the interviewer (AOR = 2.32, CI = 1.06–5.08, P = 0.015). Moreover, there was a significant interaction between education and interviewer (AOR = 3.69, CI = 1.33–10.25, P = 0.01), indicating that one field guide was much more successful in convincing individuals to use ITNs. In the multivariate model that includes the interaction between field guide and education group, the effect of the education intervention was greater for the male interviewer (AOR = 3.89) than for the female interviewer (AOR = 2.44). Of note, the adjusted effect of the educational intervention on individual ITN use with either interviewer is greater than the unadjusted effect (OR = 1.9).

### Malaria knowledge assessment

Pre-intervention malarial disease recognition was high (Table 2). Most individuals could identify malaria as the most common disease in their village (93%), recognise malaria based on clinical symptoms (98%), were familiar with the term malaria in their local language (99%) and state at least one symptom of the disease (93%). Knowledge about who was most susceptible to malaria (72%) and malaria treatment methods (87%) was also relatively high. However, knowledge of prevention was more limited. Only 35% of individuals knew that malaria was transmitted by mosquitoes and less than 40% of people knew that one could prevent malaria. Only 17% of those individuals stated that using ITNs was an important method of prevention.

**Table 2:** Pre- and post-intervention levels of knowledge about malaria for all individuals in Piron, Mali.*

	**Education group**		**Control group**	
	**Pre-intervention**	**Post-intervention**	**Pre-intervention**	**Post-intervention**

**Question**	**N (%)**	**N (%)**	**N (%)**	**N (%)**

1. What is the most common disease in your village?	137 (93)	147 (100)	115 (93)	124 (100)
2. If you or your child has the following symptoms: fever, headache, vomiting and chills, what is the disease?	144 (98)	147 (100)	121 (98)	124 (100)
3. Do you know what malaria is?	145 (99)	146 (99)	122 (98)	124 (100)
4. Name at least one symptom of malaria.	139 (95)	147 (100)	112 (90)	124 (100)
5. Who is most susceptible to malaria?	53 (36)	130 (88)	41 (33)	107 (86)
6. How is malaria transmitted?	111 (76)	131 (89)	84 (68)	118 (95)
7. Can you prevent malaria?	53 (36)	131 (89)	46 (37)	102 (82)
8. How can you prevent malaria?	49 (33)	131 (89)	47 (38)	102 (82)
9. Can you treat malaria?	132 (90)	147 (100)	111 (90)	122 (98)
10. How can you treat malaria?	129 (88)	147 (100)	107 (86)	123 (99)
**Total knowledge score ** **(Maximum 10 points) **†	**7.43 ± 1.85**	**9.55 ± -.96**	**7.31 ± 1.88**	**9.44 ± 1.06**

*One point was given for any correct answer, 0 points was given for an incorrect answer, and one point was subtracted for any incorrect answer for questions with multiple answers. This method of scoring responses produced a normal distribution when assessing the change in knowledge for individuals from baseline to post-intervention surveys. Correct answers to the questions are: 1. Malaria, 2. Malaria, 3. Yes, 4. Fever, headache, chills, yellow eyes, vomiting, diarrhoea, 5. Pregnant women, children, 6. Mosquito bite, 7. Yes, 8. Insecticide-treated net (ITN), untreated net, modern medicine, traditional medicine, 9. Yes, 10. Modern medicine, traditional medicine. All pre- and post-intervention differences were significant (P < 0.001) except for questions two and three. †Plus-minus values are means ± standard deviations (SD).

Within subject, change in knowledge score pre- versus post-educational intervention was also significant (average change score = 2.13, standard deviation = 1.97, t = -17.78, P < 0.001). However, there was no significant difference in responses to individual questions or in total knowledge score between the group which received the educational component and the control group when analysed by household (education group change score = 2.14 vs. control group change score = 2.12 P = 0.96) or by individual (education group change score = 2.12 vs. control group change score = 2.13 P = 0.98). When household analysis was adjusted for baseline characteristics, none affected the impact of the intervention on knowledge scores. When the analysis was adjusted for time of interview, it was found that individuals (and households) who were interviewed later during the intervention period had significantly lower changes in knowledge score (B = -0.34, CI = -0.56 to -0.11, P = 0.004).

## Discussion

The study results suggest that education increases net-treatment rates. Antecedent education was significantly associated with ITN use at the individual level, but not at the household level. Additionally, knowledge about malaria increased overall for the entire village, but no significant difference was found between education and control groups.

Other studies have found that ITN use increased when individuals received health promotional activities about ITNs [15,16], although few studies have examined the effects of ITN educational interventions in a control trial. There are several reasons that could explain why a difference was not found at the household level. First, the power of our study to detect differences between intervention and control households may have diminished over time as information diffused from one household to another. In fact, households that were interviewed during the first two days of the study were more likely to impregnate their nets if they received the educational intervention compared to control. Second, simply being in the village may have had an immeasurable influence on net treatment rates. Almost everyone had heard about ITNs previously, but was not using them. Taking time with each individual and/or household to offer net impregnation services from an 'expert' source may have been sufficient to change behaviour. Finally, any ITN use in a household use is a conservative estimate of total ITN use because most households owned more than one net and many people often share a net. Of note, the analysis of ITN use had more power to detect a difference between intervention and control groups, and the effect remained significant after adjusting for within household correlation.

Another important objective of this study was to install net-impregnation services within the village run by community members themselves so that accessibility did not prevent ITN use. A previous study done in Mali demonstrated that individuals who used ITNs were predominantly from communities that had net-treatment services in their village [13]. Other studies have also shown that community involvement is an important factor to the success of net-impregnation programs [12,17,18].

Approximately 40% of the village impregnated their nets, which illustrates that availability of services and education can increase ITN use, but there remain substantial barriers to achieving the NMCP goal of 90% use. Cost is clearly a factor as individuals who made a monetary income were more likely to impregnate their nets. Of those who did not impregnate their nets during this study, 93% of individuals said that cost was the main factor. Similar results have been found in other studies [10,19,20]. However, the socio-economic data collected in this study suggest that many individuals were able to afford the promotional price of treating one net. Guigemde *et al*. have shown that treating nets with insecticide is affordable to many individuals in malaria-endemic areas, but they are not aware of how much they spend on other, often less effective, prevention methods [21]. Individuals may also need time to see that impregnation is more effective than untreated nets alone. It was found that more individuals impregnated their nets at later time points during the promotional service once they had seen others benefit from ITN use.

There are limitations to the study. The biggest shortcoming is that the study was done within one village, thus placing control and intervention groups in close proximity. There is a strong possibility that knowledge diffused between individuals who received the educational component and those individuals who did not, as suggested by the fact that almost everyone knew more about malaria after the education intervention. A more rigorous study design would use several widely separated villages rather than households within the same village. However, the limited time and resources precluded identifying and implementing the project in multiple villages with comparable demographic factors.

Second, the field guide who administered the questionnaire and provided the education affected the impact of the education intervention on ITN use, as was demonstrated by the interaction between education and field guide. There is no reason to believe that the educational intervention was delivered differently by each field guide, as both were given the same training and tested on the reproducibility of their presentations. One field guide may simply be a better salesman in promoting net treatment. Regardless of field guide influence, individuals receiving education were still significantly more likely to impregnate their bednets than control group individuals, as shown by the fully adjusted analysis.

Third, there was limited power in this study to detect small effect sizes. The study was planned to have sufficient power to detect a 50% increase in ITN use given the size of the village. The number of households required to detect the effect size observed in this study (400) is larger than the village itself. As mentioned above, resources were not sufficient to include other villages.

Finally, sustainability of net-impregnation program in the long-term was not determined during this study. Although there was not complete ITN use in Piron at the conclusion of the study, increased ITN use may be seen in Piron after the rainy season has finished when malaria transmission is heaviest and the advantages of ITNs are more pronounced. A follow-up study is needed in order to see if this is correct.

## Conclusion

Despite potential limitations, the data suggest that it is possible to educate individuals about malaria and to implement net impregnation services in villages with limited resources. ITNs are currently one of the most viable options for reducing malaria-related morbidity and mortality. Although ITN use is the primary method recommended by the World Health Organization for malaria reduction and control, implementation worldwide has been slow. Some of the major barriers to ITN use are lack of access determined by locality of services, misunderstanding the costs in relation to the benefits of ITNs and low levels of knowledge about malaria prevention. Greater accessibility to net-impregnation services is necessary but not sufficient to increase ITN use. The present study evaluated a project to simultaneously increase access to ITN and malaria prevention knowledge using a controlled study design. This study also illustrates the difficulties associated with behaviour change intervention research. More studies such as this one, which combines program delivery using minimal resources with evidence-based evaluation to address major local health concerns in developing countries, need to be done.

## Authors' contributions

MR participated in the design and coordination of the study, carried out the field work and data collection, performed statistical analysis of the data collected and drafted the manuscript. MS participated in the design and coordination of the study and contributed to the writing of the manuscript. SP participated in the design of the study, performed statistical analysis of the data collected and contributed to the writing of the manuscript. WM participated in the design of the study and contributed to the writing of the manuscript. JP participated in the design of the study and contributed to the writing of the manuscript. OD participated in the design and coordination of the study and contributed to the writing of the manuscript.
